# Increased Response to Glutamate in Small Diameter Dorsal Root Ganglion Neurons after Sciatic Nerve Injury

**DOI:** 10.1371/journal.pone.0095491

**Published:** 2014-04-18

**Authors:** Kerui Gong, Ling-Hsuan Kung, Giulia Magni, Aditi Bhargava, Luc Jasmin

**Affiliations:** 1 Department of Oral and Maxillofacial Surgery, University of California San Francisco, San Francisco, California, United States of America; 2 Department of Anatomy, University of California San Francisco, San Francisco, California, United States of America; 3 Department of Surgery, University of California San Francisco, San Francisco, California, United States of America; 4 Osher Center for Integrative Medicine, University of California San Francisco, San Francisco, California, United States of America; Boston Children's Hospital and Harvard Medical School, United States of America

## Abstract

Glutamate in the peripheral nervous system is involved in neuropathic pain, yet we know little how nerve injury alters responses to this neurotransmitter in primary sensory neurons. We recorded neuronal responses from the *ex-vivo* preparations of the dorsal root ganglia (DRG) one week following a chronic constriction injury (CCI) of the sciatic nerve in adult rats. We found that small diameter DRG neurons (<30 µm) exhibited increased excitability that was associated with decreased membrane threshold and rheobase, whereas responses in large diameter neurons (>30 µm) were unaffected. Puff application of either glutamate, or the selective ionotropic glutamate receptor agonists alpha-amino-3-hydroxy-5-methyl-4-isoxazolepropionic acid (AMPA) and kainic acid (KA), or the group I metabotropic receptor (mGluR) agonist (*S*)-3,5-dihydroxyphenylglycine (DHPG), induced larger inward currents in CCI DRGs compared to those from uninjured rats. N-methyl-D-aspartate (NMDA)-induced currents were unchanged. In addition to larger inward currents following CCI, a greater number of neurons responded to glutamate, AMPA, NMDA, and DHPG, *but not* to KA. Western blot analysis of the DRGs revealed that CCI resulted in a 35% increase in GluA1 and a 60% decrease in GluA2, the AMPA receptor subunits, compared to uninjured controls. mGluR1 receptor expression increased by 60% in the membrane fraction, whereas mGluR5 receptor subunit expression remained unchanged after CCI. These results show that following nerve injury, small diameter DRG neurons, many of which are nociceptive, have increased excitability and an increased response to glutamate that is associated with changes in receptor expression at the neuronal membrane. Our findings provide further evidence that glutamatergic transmission in the periphery plays a role in nociception.

## Introduction

Glutamate is increasingly recognized as a nociceptive neurotransmitter in the periphery [1]. Glutamate receptors (GluRs) in the soma of primary sensory neurons are exported to the nerve terminals in the skin, muscles and joints [2], [3], [4], [5], [6], [7], [8], [9], [10]. Given that a large proportion of GluR bearing peripheral fibers are unmyelinated, peripheral glutamatergic transmission is believed to be involved in nociceptive transmission. Notably, 47% of unmyelinated peripheral axons are immunopositive for N-methyl-D-aspartate (NMDA) receptors and 28% for kainate (KA) receptors [11]. With peripheral inflammation these receptors become sensitized and the number of peripheral axons immunopositive for GluRs increases [11], [12]. When glutamate is administered directly in the sensory ganglion it can both trigger action potentials and sensitize neurons to incoming potentials, in an NMDA-receptor dependent fashion [13]. Blocking NMDA, AMPA, kainate, and mGluR group I receptors in peripheral tissues attenuates pain behavior and activity of nociceptive sensory neurons in inflammatory or neuropathic models [8], [14], [15], [16], [17], [18], [19].

Our own work and recent work by Laursen and colleagues on the nociceptive effects of glutamatergic transmission in the periphery has focused on the sensory ganglion, where altering local glutamate uptake or recycling led to changes in nociceptive behavior [13], [20], [21]. We also found that glutamate expression increases in the soma of dorsal root ganglion (DRG) sensory neurons following peripheral nerve injury [6]. These observations lead us to postulate that glutamate neurotransmission occurs within the sensory ganglion [6] and that functional GluRs are expressed at the somatic surface of primary sensory neurons in the DRGs. Just as in the terminals [11], GluRs might become sensitized and show changes in their expression after peripheral injury. To test our hypothesis, patch clamp recordings were done on *ex-vivo* preparations of whole DRGs from rats with seven days of a chronic constriction injury (CCI) of the sciatic nerve. Small (<30 µm) and large (>30 µm) diameter neurons from L4 and L5 DRGs were used to record inward currents and rheobase from naïve and rats with CCI. Agonists to ionotropic GluRs and group I metabotropic GluRs (mGluRs) were puff-applied in the vicinity of the neuronal membrane. As groups II and III mGluRs are known to be inhibitory and do not induce measurable currents [22], the role of these receptors was not investigated in the present study.

Paw inflammation is reported to cause changes in GluRs expression in peripheral axons [11], thus, in our injury model we also determined if GluRs expression was changed using western blot. We specifically monitored the expression of the AMPA receptor GluA1 and GluA2 subunits as well as that of group I mGluRs because of their known association with neural plasticity. Given that surface expression of GluRs is closely linked with neuronal excitability [23], we also determined changes in the expression of *membrane-bound* receptors after nerve injury. The results show that peripheral injury is accompanied by an increased membrane distribution of the intraganglionic GluRs.

## Materials and Methods

### Animals

Male Sprague-Dawley rats (180–200 g) were housed on a 12-hour light–dark cycle and given food and water *ad libitum*. For electrophysiological recordings, we used 12–20 animals/group. For western blot analysis, DRGs (L4 and L5 pooled from each animal) ipsilateral to the injured nerve with 5–8 animals/group were used.

### Ethics

Procedures for the maintenance and use of the experimental animals conformed to the regulations of UCSF Committees on Animal Research and were carried out in accordance with the guidelines of the NIH regulations on animal use and care (Publication 85–23, Revised 1996). The UCSF Institutional Animal Care and Use Committee approved the protocols used in this study.

### Chronic Constriction Injury (CCI) Surgery

CCI was performed as described previously [24] on rats under isoflurane anesthesia (2%, Solvay, Mendota Height, MN USA). Briefly, the sciatic nerve was exposed at the level of middle thigh and four 4-0 loose chromic gut ligatures (Ethicon, Somerville, NJ USA) were loosely tied proximal to the trifurcation of the sciatic nerve. In all cases, care was taken not to put tension on the nerve, or its branches. Muscle and skin were closed in two layers and the rats were returned to their cages. Rats were euthanized seven days after CCI.

### Intact dorsal root ganglion (DRG) preparations and whole cell patch clamp recording

For preparation of intact DRGs, rats were deeply anaesthetized with sodium pentobarbital (40 mg/kg, i.p.). A laminectomy was performed, and the L4 and L5 DRG with about 20 mm of attached dorsal root and 15 mm of spinal nerve were removed and placed into artificial cerebral spinal fluid (aCSF) bubbled with carbogen. The aCSF contained: 124 mM NaCl, 2.5 mM KCl, 1.2 mM NaH_2_PO_4_, 1.0 mM MgCl_2_, 2.0 mM CaCl_2_, 25 mM NaHCO_3_ and 10 mM glucose. The connective tissue surrounding the DRG was carefully removed under a dissecting microscope, and the ganglion was transferred to a recording chamber through which aCSF was perfused at a rate of 2–3 mL/min. A fine mesh anchor (SHD-22L, Harvard, USA) was used to stabilize the DRGs during recording. Five unit/mL of liberase TM (Roche) was applied locally via a pipette with a 5 µm diameter tip. After 15–20 minutes, the digested epineurium residue was cleaned to expose the neurons.

DRG neurons were visualized with a 40X water-immersion objective using a microscope (FN-600; Nikon, Japan) equipped with infrared differential interference contrast optics. The image was captured with an infrared-sensitive CCD (IR-1000, Dage MTI, USA) and displayed on a black-white video monitor. Currents were recorded with an Axon 200B amplifier (Molecular Devices, USA) connected to a Digidata interface (Digidata 1322A, Molecular Devices, USA) and low-pass filtered at 5 kHz, digitized, and stored using pCLAMP 10.2 (Molecular Devices, USA). Patch pipettes were pulled from borosilicate glass capillary tubing (BF150-86-10, Sutter, USA) with a P97 puller (Sutter, USA). The resistance of the pipette was 4–5 MΩ when filled with recording solution, which contained: 140 mM KCl, 2 mM MgCl_2_, 10 mM HEPES, 2 mM Mg-ATP, 0.5 mM Na_2_GTP, pH 7.4. Osmolarity was adjusted to 290–300 mOsm. After a gigaseal was established, the membrane was broken and neurons were selected for further study if they had a resting membrane potential less than −50 mV. The access resistance was 10–20 MΩ and was continuously monitored. Data were discarded if the access resistance changed more than 15% during an experiment. For measuring the rheobase, a series of currents was injected to the neuron, starting at −0.1 nA with increments of 0.05 nA until the first action potential was generated. For measuring the membrane threshold, a 500 ms depolarizing ramp (2000 pA/s) was administered. For all currents induced by agonists except NMDA, the neurons were clamped at −70 mV. For NMDA recordings, the neurons were clamped at −40 mV, and Mg^2+^-free aCSF was used to remove the blocking effect of magnesium.

### Drug application

All drugs were purchased from Tocris (Bristol, UK). Drugs were dissolved in ultra-pure deionized water as stock solutions. All stock solutions were diluted to the desired concentration with aCSF immediately before use. Glutamate (1 mM), AMPA (100 µM), NMDA (100 µM), KA (100 µM) and DHPG (1 mM) were applied with focal pressure ejection via a puffer pipette controlled by a Picrospitzer II (200 ms puff at 1-2 psi, General Valve, USA) to activate the receptors. The pipette was located approximately 50 µm from the recorded neuron so that the drugs reached all parts of the neuron. Changes in currents that were greater than 20% of baseline were determined to be inward currents induced by the agonists. We used both 100 µM and 1 mM concentration of DHPG for their ability to induce inward currents. While both concentrations induced robust inwards currents with similar amplitudes, the data with 1 mM was more consistent than with 100 µM. We attributed that to the fact that 1 mM may be more effective in saturating all cell surface receptors, thereby giving a more consistent data, thus results obtained using 1 mM DHPG are reported here. The antagonists, APV (50 µM), CNQX (10 µM), and DL-AP3 (60 µM) were bath-applied for at least 5 minutes to test the blocking effect; importantly, no rundown phenomena were observed during the 5 min incubation period. For DHPG treatment, DRGs were incubated in a vial with 50 µM DHPG for 2 hours.

### Western blot

DRGs pooled from L4+L5 (from 5–8 animals) were homogenized in lysis buffer (30 mM Tris HCl, 1 mM EGTA, 0.1 mM Na_3_VO_4_, 10 mM Na_4_P_2_O_7_, 10 mM NaF, pH 7.4) containing complete protease inhibitor mini EDTA-free (Cat#1835170, Roche Diagnostics) and phosphatase inhibitor cocktail (Cat # P0044, Sigma-Aldrich). Tissue homogenates were centrifuged at 23,000 g for 10 minutes at 4°C. The supernatant contained the cytosolic fraction. To obtain the membrane-bound fraction, the pellets were re-suspended in the lysis buffer containing 1% triton X-100. Samples were sonicated for 1 minute (5 second pulses) and centrifuged at 23,000 g for 15 minutes at 4°C. The resultant supernatant contained the membrane-bound protein fraction. BCA assays were performed to determine the protein concentration in each membrane or cytosolic fraction. For western blot analysis, 20 µg of protein was separated on a 10% SDS-PAGE and transferred onto PVDF membranes as described by us previously [25]. Membranes were blocked in Blocking Buffer (LI-COR Biosciences) for 1 hour at room temperature and incubated overnight at 4°C with primary antibodies: monoclonal mouse anti-β-actin (1∶7000, cat#: A2228, Sigma-Aldrich), mouse anti-N-cadherin (1∶1000, Cat #610920, BD Biosciences), rabbit polyclonal anti-GluA1 (1 µg/ml, Cat #AB1504, Millipore), monoclonal mouse anti-GluA2 (1∶200, Cat#75-002, NeuroMab Clone L21/32, UC Davis/NIH NeuroMab Facility, USA), monoclonal mouse anti-mGluR1α (1∶1000, Cat #556389, BD Biosciences) and polyclonal rabbit anti-mGlu5 (1∶2000, Cat #AB5675, Millipore) diluted in the blocking buffer. Membranes were washed in TBS-T (TBS plus 0.1%Tween20), incubated for 1 hour at room temperature with fluorescent secondary (IRDye 680RD polyclonal donkey anti-mouse IgG Cat #926-68072, or IRDye 800CW polyclonal goat anti-rabbit IgG Cat #926-32211), washed, imaged, and quantified using the LI-COR Odyssey Infrared Imaging System. Proteins in the membrane fraction were normalized to N-cadherin (Fig. S1 and Tables S1–S4) and the cytosolic proteins were normalized to β-actin. No β-actin was detected in the membrane fractions and no N-cadherin was detected in the cytosolic fraction, confirming that our method is satisfactory in separating membrane fraction from the cytosolic (Fig. S2). The data shown for all receptor subunits represents normalized membrane fractions divided by sum of normalized membrane and cytosolic proteins (total normalized protein) for each receptor.

### Immunofluorescence

Rats were transcardially perfused with 4% paraformaldehyde (PFA) and lumbar dorsal root ganglia were removed, post-fixed in 4% PFA/30% sucrose and embedded in OCT compound (Tissue-Tek, Sakura Finetek). Longitudinal sections (10 µm) of DRGs were cut on a cryostat. The following primary antibody and dilution was used: monoclonal mouse anti-GluR1 1∶1000 (MAB2263, Millipore, Billerica, MA, USA). Sections were washed and incubated with species-specific CY3 secondary antibody (1∶500). Sections were visualized using Nikon Eclipse E800 epi-fluorescence microscopy and images were captured using AxionVsion (Zeiss) software (Fig. S3).

### Statistical analysis

All results were presented as the mean ± SEM. For testing the blocking effect of receptor antagonist, the responses induced by each agonist were set as 100%, and the currents after antagonist application were expressed as the percentage of previous response. The statistical significance was determined using the Student's t-test. The level of p<0.05 was assumed as statistically significant.

## Results

### CCI increased-excitability in small diameter DRG neurons, but not in large diameter neurons

**Figure 1:**
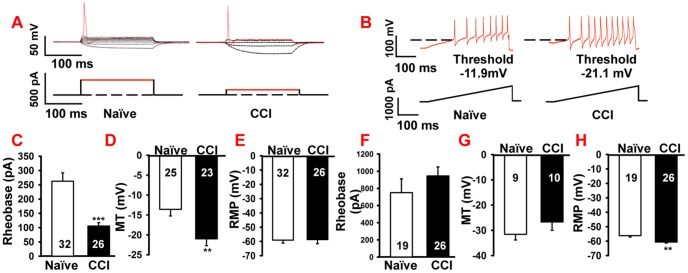
CCI of sciatic nerve increased small diameter neuron excitabilities, but had no effect on large diameter neurons. A. Current clamp recordings of the membrane potential (upper traces) and injected current (lower traces) from small diameter DRG neurons of naïve and CCI animals, showing a lowered rheobase after CCI. B. Following CCI, small diameter neurons also exhibited a lowered membrane threshold (upper trace) during a ramp stimulus (lower trace). The dashed lines indicate a membrane threshold of −11.9 mV for the naïve neurons and −21.1 mV for CCI neurons. Statistical analysis showed that CCI significantly reduced rheobase (C) and membrane threshold (D) compared to naïve animals. No difference was found in resting membrane potential between naïve and CCI animals (E). Large diameter neurons showed no difference in rheobase (F) or membrane threshold (G) between naïve and CCI animals, but the CCI group had a lower resting membrane potential (RMP) compared with the naïve group (H). Numbers in each column represents recorded neurons. MT: membrane threshold. Mean ± SEM, ** p<0.01, *** p<0.001.

Most DRG nociceptive neurons are small diameter neurons (<30 µm diameter) [26]. In order to determine changes in membrane properties of small diameter DRG neurons, L4 and L5 ganglia from naïve and rats with CCI of the sciatic nerve were removed and prepared for *ex-vivo* patch-clamp recordings. The rheobase for small diameter neurons in the CCI group was decreased over 60% compared with the naïve group (CCI, 105.8±11.6 pA, naïve, 262.5±29.4 pA; p<0.001, Fig. 1A and 1C). Similarly, the membrane threshold was also decreased in CCI animals compared to neurons from the naïve group (CCI, −21.1±1.7 mV; naïve, −11.2±1.6 mV; p<0.001, Fig. 1B and 1D). In contrast, the resting membrane potential (RMP) of small diameter neurons did not differ between naïve and CCI rats (naïve, −59.1±2.1 mV; CCI, −58.7±2.9 mV; p>0.05. Fig.1E). These findings show that after nerve injury, small diameter DRG neurons have an increased excitability as indicated by the average decrease in the rheobase and the membrane threshold.

Large diameter neurons (>30 µm), many of which transmit innocuous sensation, showed no differences in membrane properties between naïve and CCI rats for the rheobase (naïve, 750.3±161.8 pA; CCI, 945.1±105 pA; p>0.05, Fig.1F) or the membrane threshold (naïve, −31.5±2.3 mV; CCI, −26.6±3.1 mV; p>0.05, Fig. 1G). However, the RMP for the neurons in the CCI group showed hyperpolarization compared to neurons from naïve rats (CCI, −60.6±0.7 mV; naïve, −56.2±0.9 mV; p<0.01, Fig. 1H). Taken together, these data suggested to us that CCI injury decreased, rather than increased, neuronal excitability in large diameter neurons.

### Glutamate receptor-mediated inward currents are increased following CCI

**Figure 2:**
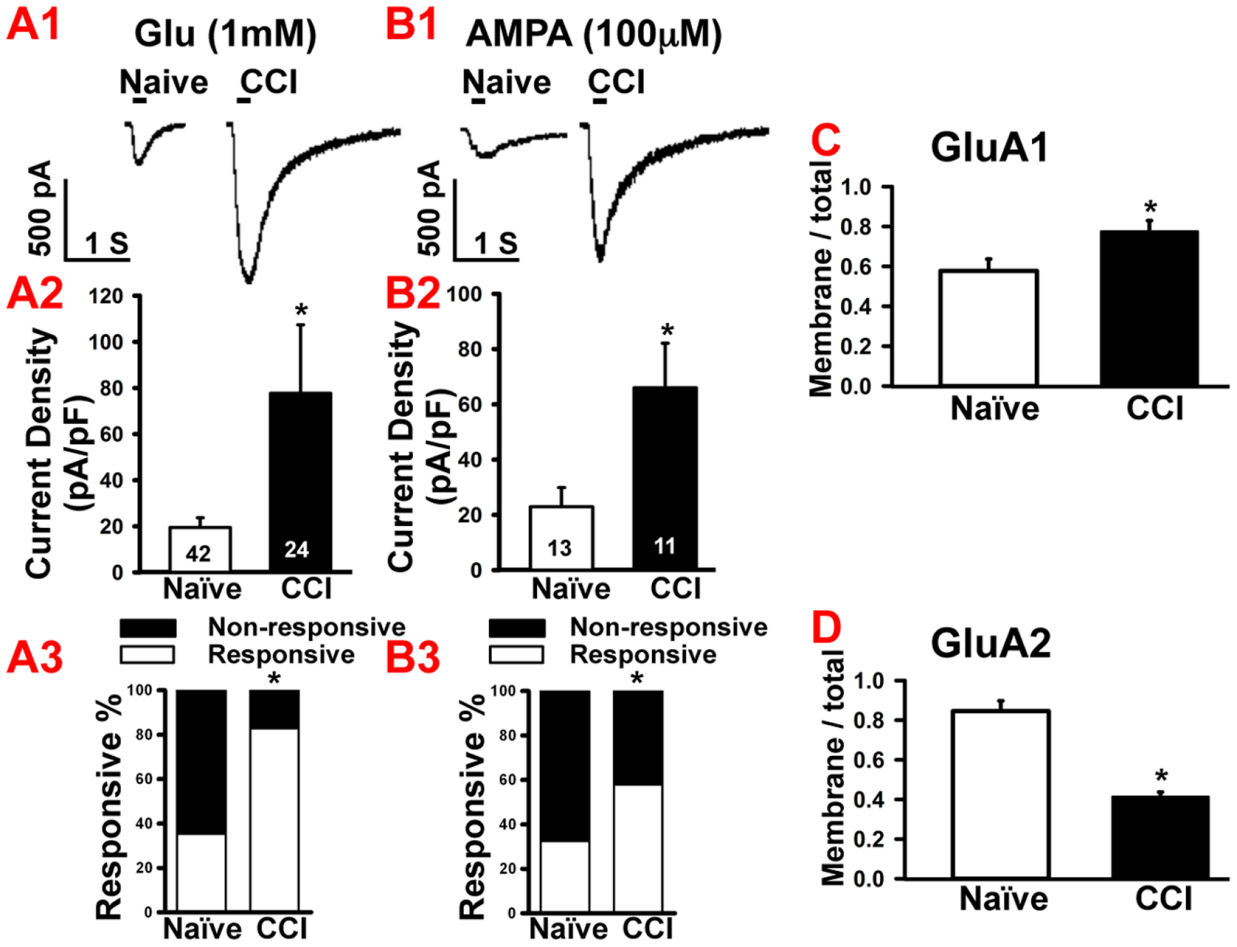
CCI of sciatic nerve increased responses to glutamate and AMPA in small diameter neurons. A1. Example of glutamate-induced (1 mM, 200 ms) increases in inward currents in small diameter neurons after CCI compared to naïve. Bar indicates the duration of drug application. A2. Statistical analysis showed that glutamate induced significantly larger inward currents in the CCI group compared with naïve. A3. Population analysis showed that CCI increased the proportion of the neurons responsive to glutamate. B1. Example of AMPA-induced (100 µM, 200 ms) increases in inward current in small diameter neurons after CCI compared to naïve. Bar indicates the duration of drug application. B2. Statistical analysis showed that AMPA induced significantly larger inward currents in the CCI group compared with naïve. B3. Population analysis showed that CCI increased the proportion AMPA-responsive neurons. C. Western blot analysis showed increased GluA1 expression (normalized membrane/normalized total protein) in naïve and CCI DRGs. D. Western blot analysis showing decreased GluA2 expression in naïve and CCI DRGs. Numbers in each column represents recorded neurons. Mean ± SEM, * p<0.05, ** p<0.01.

Having established that excitability of non-nociceptive large diameter neurons was unaltered after CCI, we focused on the responses of small diameter neurons to glutamate after injury. To investigate the activity of glutamate receptors on the neuronal soma, we examined the response of DRG neurons to puff application of glutamate in naïve animals and found that 35.3% neurons (42/119) responded with inward currents. The amplitudes of inward currents ranged from 11.2 to 1221.3 pA, with a mean current density of 19.54±4.2 pA/pF (Fig. 2A1 and A2). To confirm that the inward currents were mediated via stimulation of glutamate receptor, we bath-applied a mixture of 50 µM APV, a NMDA receptor antagonist and 10 µM CNQX, an AMPA/kainate receptor antagonist for 10 min prior to glutamate puff application. The glutamate-induced currents decreased to 15.8% of the original response (n = 7, 15.8±3.4% of pre-drug response, p<0.01) after glutamate receptor antagonists.

After CCI of the sciatic nerve, 82.8% (24/29, p<0.05) of the neurons in the L4 and L5 ganglia responded to glutamate puff application (Fig. 2A3). The mean current density in glutamate-responsive neurons increased to an average value of 77.7±29.7 pA/pF compared with 19.54±4.2 pA/pF in naïve neurons (Fig. 2A1 and 2A2, p<0.05). Further analysis showed that after CCI, 40% of the glutamate-responsive neurons showed inward currents larger than 1000 pA, in contrast to neurons from the naïve rats, where only 2.9% had responses larger than 1000 pA.

Next, in order to determine which specific glutamate receptor subunits contributed to the increased currents after nerve injury, we examined the responses of naïve and CCI ganglia to AMPA, NMDA, KA, and group I mGluRs agonists.

### CCI increases AMPA receptor-mediated inward currents and the percentage of AMPA-responsive small diameter neurons

In ganglia from naïve rats, 32.5% (13/40) small diameter neurons responded to puff application of AMPA (100 µM), whereas following CCI, the number of AMPA-responsive neurons increased to 57.9% (11/19). The mean current density in the naïve group was 23.0±6.9 pA/pF and 66.0±16.1 pA/pF in the CCI group (Fig. 2B1 and B2), which was an increase of ∼187% (p<0.01, naïve vs. CCI). Bath application of 0.1 µM of CP465022, an AMPA selective receptor antagonist, blocked the AMPA-induced currents to 5.8±0.9% of the original response (data not shown), confirming that the currents were mediated by AMPA receptors in a subpopulation of small diameter neurons.

We next determined whether the increased AMPA currents seen after CCI were due to increased presence of AMPA receptor subunits, GluA1 and GluA2 at the membrane. We used western blot analysis to compare the ratio of normalized membrane-bound receptor subunits to total AMPA receptor (membrane + cytosolic) from naïve and CCI DRGs. CCI resulted in a 35% increase in GluA1 receptor subunit in the membrane fraction (CCI 0.77±0.05; naïve, 0.57±0.05; p<0.05, Fig. 2C), whereas CCI decreased membrane GluA2 expression by 51.8% (CCI, 0.41±0.02; naïve, 0.85±0.05; p<0.01, Fig. 2D).

### CCI did not change NMDA-induced inward currents in small neuron, but increased the percentage of NMDA- responsive neurons

**Figure 3:**
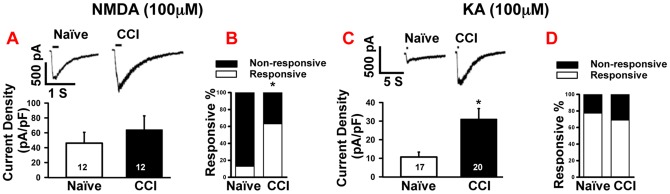
CCI of sciatic nerve increased responses to NMDA and KA in small diameter neurons. A. Upper panel: Puff application of NMDA (100 µM, 200 ms) had no effect on inward currents in small DRG neurons from naïve or CCI rats. Bar indicates the duration of drug application. Lower panel: Statistical analysis showed no differences in inward currents induced by NMDA between CCI and naïve rats. B. Population analysis showed that the percentage of the NMDA-responsive neurons increased after CCI. C. Upper panel: Example of KA-induced (100 µM, 200 ms) increases in inward currents in CCI DRGs. Bar indicates the duration of drug application. Lower panel: Statistical analysis showed that KA induced a significantly larger response in CCI group compared with naïve. D. Population analysis showed that the percentage of the KA-responsive neurons did not change significantly after CCI. Numbers in each column represents recorded neurons. Mean ± SEM, * p<0.05.

To measure the neuronal responses to NMDA, small diameter neurons were clamped at −40 mV, and incubated in Mg^2+^-free aCSF to remove the magnesium block. Puff application of 100 µM NMDA significantly increased the percentage of NMDA-responsive neurons from 21.8% (12/55) in naïve ganglia to 70.6% (12/17) in CCI ganglion (p<0.05. Fig.3B). Surprisingly, despite the increase in the number of NMDA-responsive neurons, there was no significant difference in the mean current density of NMDA-induced inward currents between the naïve and CCI groups (naïve, 46.2±14.5 pA/pF; CCI, 64.0±18.7 pA/pF; p = 0.46, Fig. 3A). To ascertain the contribution of NMDA on inward currents, 50 µM of APV, a selective NMDA antagonist, was bath-applied on a subset of neurons from naïve ganglion. Following APV application, the mean amplitude of NMDA-responsive inward currents was reduced to 13.0%±4.0 of the original response (pre-APV, 31.2±10.1pA/pF; post-APV 4.0±1.5 pA/pF; p<0.01, data not shown).

### CCI enhanced KA receptor-mediated inward currents in small diameter neurons

The mean amplitude of KA-induced inward currents in the CCI group was increased to over 500% compared with the naïve group (CCI, 792.3±193.2 pA; naïve, 128.5±32.0 pA; p<0.01, Fig. 3C). In naïve rats, 77.3% (17/22) of the small diameter neurons responded to puff application of 100 µM KA, which was the largest number for any agonist in the naïve group. In CCI ganglia, 69% (20/29) of neurons were KA-responsive and similar percent of neurons were KA-responsive in the naïve group (Fig. 3D; p>0.05). In a subgroup of neurons from the naïve group, bath application of 0.5 µM UBP310, a KA specific antagonist dramatically blocked the currents induced by puff application of KA (pre-UBP310, 203.9±73.7 pA, n = 6; post-UBP310, 5.1±1.3 pA, n = 6; p<0.001, data not shown).

### CCI enhanced group 1 mGluR-mediated inward currents in small diameter neurons

**Figure 4:**
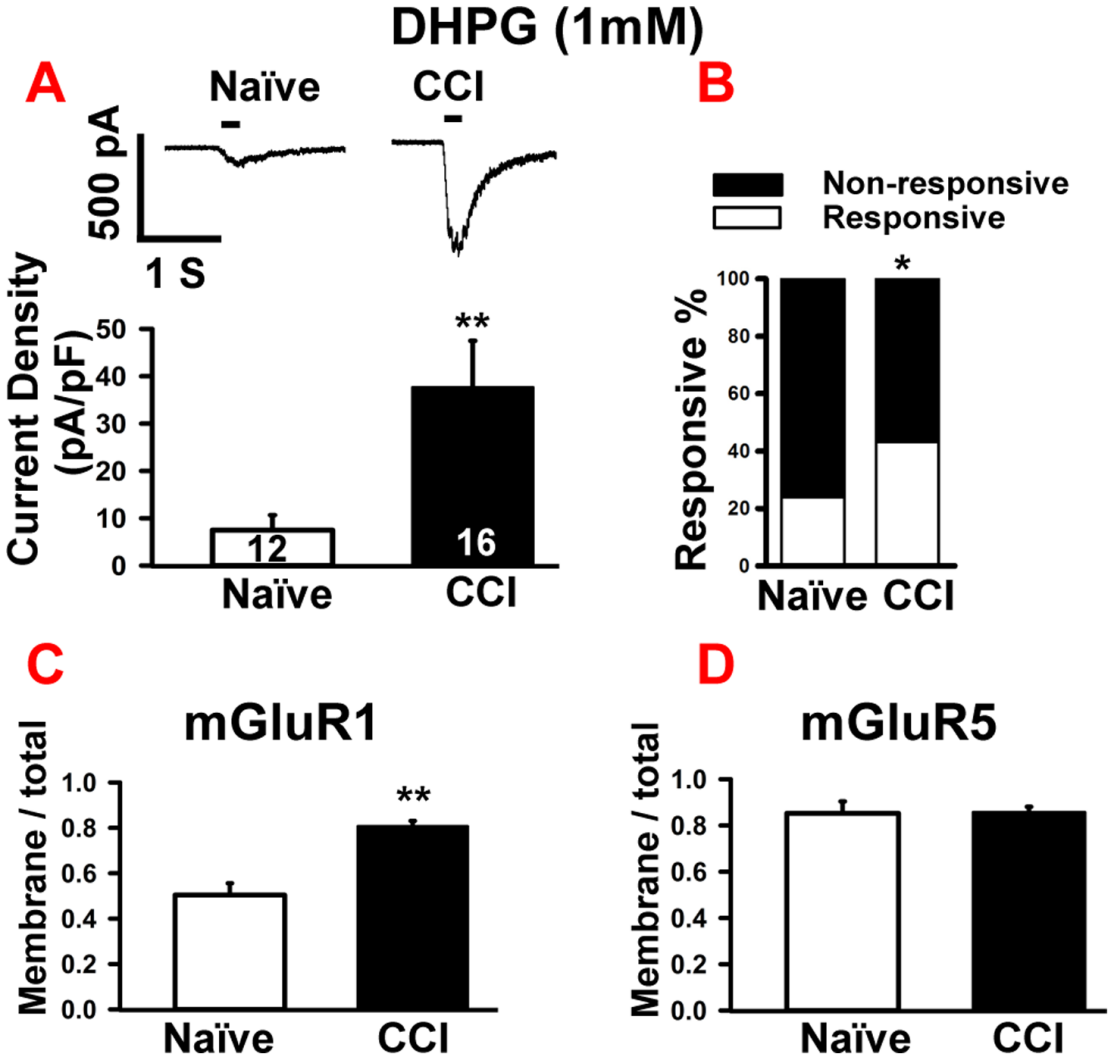
CCI of sciatic nerve increased responses to DHPG in small diameter neurons. A. Upper panel: Example of DHPG-induced (100 µM, 200 ms) increases in currents in CCI vs. naïve DRGs. Bar indicates the duration of drug application. Lower panel: Statistical analysis showed that DHPG induced a much bigger response in CCI group compared with naïve. B. Population analysis showed that the percentage of DHPG-responsive neurons increased after CCI. C. Western blot analysis showed increased mGluR1 expression (normalized membrane/normalized total protein) in naïve and CCI DRGs. D. Western blot analysis showing that the expression of mGluR5 subunit in the membrane did not change significantly after CCI, compared to the naïve group. Numbers in each column represents recorded neurons. Mean ± SEM, * p<0.05, **p<0.01.

Finally, we tested changes in response of mGluRs with the selective group I agonist DHPG. Bath application of 1 mM DHPG increased the percentage of responsive neurons from 24% (n = 12) in the naïve group to 43.2% (n = 16) in the CCI group (p<0.05, Fig. 4B). As with the other glutamate receptor agonists, the mean current density of inward currents for DHPG in the CCI ganglion was increased, compared to the naïve (CCI, 37.7±9.8 pA/pF, naïve, 7.5±3.2 pA/pF; p<0.05, Fig. 4A). Bath application of 50 µM of DL-AP3, a group I mGluR selective receptor antagonist, significantly reduced the amplitude of DHPG-induced inward currents to 16.3%±5.1 of the original response in a subset of the naïve group neurons (n = 7; p<0.001, data not shown), confirming that the currents induced by the DHPG were mediated by group I mGluR.

DHPG can mediate its action via both mGluR1 and mGluR5 and as there is no selective agonist for each of the two subunits, we therefore used western blot analysis to determine whether receptor levels of mGluR1 and mGluR5 were altered following CCI. Using well-characterized antibodies that can differentiate between the two-receptor subunits, we found that CCI injury resulted in ∼60% increase in mGluR1 expression in the membrane fraction (CCI, 0.8±0.04; naïve, 0.5±0.05; p<0.01, Fig. 4C), whereas mGluR5 membrane expression remained unchanged (Fig. 4D). Thus, the increases in amplitude of inward currents induced by DHPG in CCI rats are in part mediated by increased membrane expression of mGluR1.

### DHPG incubation changed the inwards currents to ionotropic glutamate receptor agonists

**Figure 5:**
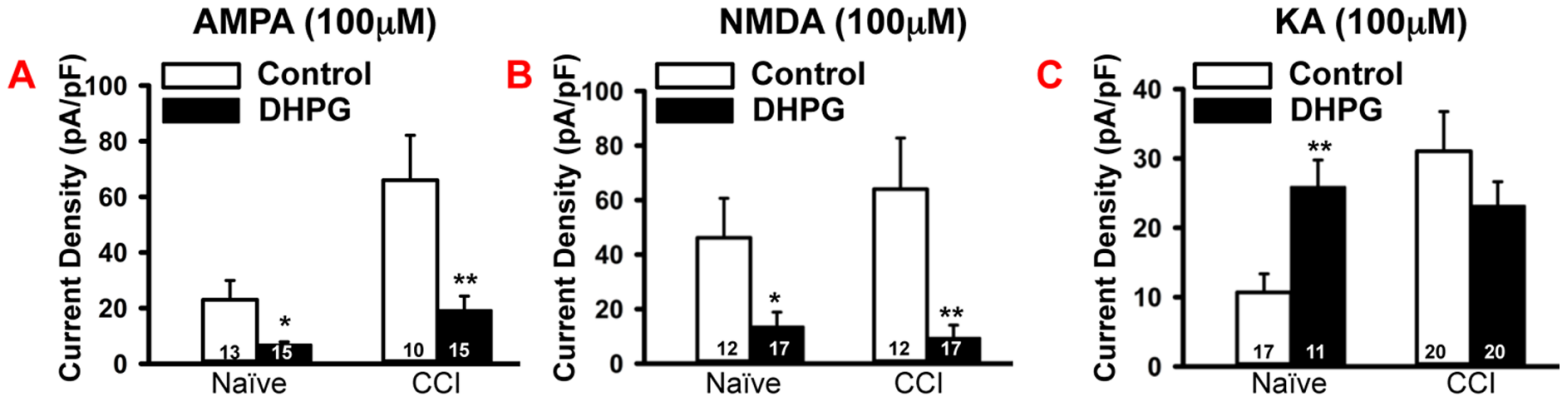
The effect of DHPG incubation on responses to AMPA, NMDA and KA in small diameter neurons. A. Incubation with 50 µM DHPG for 2 hours reduced AMPA-induced (100 µM) inward currents in small diameter neurons in both naïve and CCI groups. B. Incubation with 50 µM DHPG for 2 hours reduced NMDA-induced (100 µM) inward currents in small diameter neurons in both naïve and CCI groups. C. Incubation with 50 µM DHPG for 2 hours increased KA-induced (100 µM) inward currents in small diameter neurons in the naïve group, but had no effect on KA-induced currents in the CCI group. Numbers in each column represents recorded neurons. Mean ± SEM, * p<0.05, **p<0.01.

Since it has been shown that group I mGluR can modulate other ionotropic glutamate receptors in hippocampus and striatum [27], [28], we hypothesize that activation of group I mGluR should have similar effect in DRGs. Therefore, we incubated DRGs from naïve and CCI groups with DHPG for 2 hours. In both groups, pre-incubation of DRG neurons with 50 µM DHPG for 2 hours significantly reduced AMPA currents to approximately 30% of the original response (naïve, 23.0±6.9 pA/pF to 6.8±1.0 pA/pF, p<0.05; CCI group, 66.0±16.0 pA/pF to 19.2±5.1 pA/pF, p<0.01, Fig. 5A). DHPG incubation also reduced the NMDA currents in naïve DRGs to approximately 30% of original response (46.2±14.5 pA/pF to 13.6±5.3 pA/pF, p<0.05; Fig. 5B). In the DRGs from rats with CCI, DHPG further reduced the NMDA current to approximately 15% of the original response (64.0±18.7 pA/pF to 9.4±4.7 pA/pF, p<0.01; Fig. 5B). Surprisingly, DHPG pre-incubation resulted in either *increased* KA currents or had no effect. In the naïve group, DHPG incubation increased the KA currents by 142% from 10.7±2.7 pA/pF to 25.9±3.9 pA/pF (p<0.01, Fig.5C). In the CCI group, DHPG pre-incubation did not significantly change KA (31.1±5.7 pA/pF to 23.1±3.5 pA/pF; Fig. 5C).

## Discussion

The results presented here show that following peripheral nerve injury there are changes in the electrical properties of primary sensory neurons, as well as changes in increased responses to glutamate, and changes in the surface expression of glutamate receptors on those neurons. Given that these changes for the most part involve small diameter neurons, these finding are further evidence that glutamate plays a role in nociception in the peripheral nervous system.

### Methodological considerations

In this study we used intact DRGs with an attached portion of the peripheral nerve and dorsal rootlets. Our preparation differed from previous studies in several ways. We used adult rats and applied liberase locally at the site of recording to digest the epineurium, whereas previous studies utilizing intact ganglia, used young animals (1–2 weeks old), or bath-applied enzymatic digestion of the epineurium [29], [30]. We believe that our strategy of using local digestion resulted in less damage to the ganglion and improved the quality of the DRG preparation for patch clamp recordings.

Using whole ganglia also has the advantage that the neuron-glia relationship remains intact and the neuron retains its unipolar morphology with some part of its central and peripheral axon. This contrasts with dissociated neuron preparations where both the neuron-glia association and the unipolar morphology of the neurons are lost. In the dissociated preparation, the axotomy caused by enzyme digestion could increase neuronal excitability as indicated by a lower rheobase [31]. This explains why the rheobase values we recorded (250 pA in naïve animals) in our preparations are much higher than the 120 pA values reported by Zheng and colleagues [31]. Yet, in agreement with these authors, we also found that the rheobase of naïve-dissociated neurons was greater than that from nerve-injured animals.

To quantify changes in membrane-bound receptor expression by western blot analyses, we used protein homogenates from DRGs that contain a mix of large, medium, and small diameter neurons, as well as glial and immune cells. Thus, the changes in membrane proteins detected by western blot might reflect changes in all these cell types. However, immunofluorescence showed that glutamate receptors are predominantly expressed on small and medium diameter neurons [8], [32], [33], and after CCI the receptors appear to continue to be expressed predominantly on small and medium diameter neurons (Fig. S3).

### Increased glutamate currents in primary sensory neurons following nerve injury

Using receptor selective agonists, we showed that the increases in glutamate currents following nerve injury were mediated by AMPA, KA and group I mGluR receptors, but not by the NMDA receptors. In addition, the percentage of small diameter neurons that responded to glutamate, AMPA, NMDA and DHPG were increased, whereas the percentage responding to KA, were unchanged. This variation between the different glutamate receptor subunits in their response to injury underscores the different roles the receptor subunits play and indicates that primary sensory neurons may not have a uniform response to glutamate.

### KA receptor

Of the inotropic receptors, we found that KA showed the largest increase in inward currents being approximately 5-fold greater than in DRGs from naïve rats. The percentage of neurons responding to KA was the largest for any of the agonists tested here but was the same percentage in both naïve and CCI animals. In our study, the percentage of KA-responsive DRG neurons is consistent with previous patch clamp studies on dissociated neurons [34]. Compared to other ionotropic GluRs, the importance of KA receptors in nociception has only recently been recognized [35]. Electrophysiological evidence shows that in DRGs, KA receptors are found mainly on small and medium neurons [36]. Among the DRG neurons responsive to KA, about 80% are LA4 positive, a marker of small diameter nociceptive neurons [8]. Consistent with our results, others have shown that in the hind paw, inflammation induced up-regulation of KA receptor immunoreactivity and enhanced the KA receptor agonist-induced hyperalgesia [11], [37].

Although KA receptors are classified as ionotropic and activation leads to ion influx, there is evidence that KA receptors also have metabotropic functions [38], [39]. This occurs through activation of protein kinases, which modulate neuronal excitability by acting on other receptors and ion channels. The metabotropic function of KA receptor suggests that the result of the increased KA induced currents might not only be short term changes in neuronal excitability but could have longer term consequences through modulation or changes in expression of other receptors.

### AMPA receptor

AMPA receptor GluA1-positive neurons include both small and large diameter neurons [32]. Previous report has shown that 50% of GluA1- and peripherin-positive DRG neurons are peptidergic (express SP and/or CGRP) [23]. CCI increased AMPA-mediated inward currents ∼2-fold and also increased the percentage of neurons responsive to AMPA. Although KA showed the largest change in inward currents following CCI in terms of percentage, AMPA receptor activation resulted in the largest amplitude currents, which led us to propose that the AMPA receptor-mediated currents might contribute most significantly to nociception. Our finding of increased percentage of AMPA-responsive neurons meant that the nerve injury must alter the surface expression of AMPA receptors. In the uninjured adult CNS, the majority of AMPA receptors are Ca^2+^-impermeable (GluA1/GluA2 heteromers) and a minority are Ca^2+^-permeable (homomeric GluA1 AMPA receptors) [40]. Our western blot data indicates a change in the membrane-bound expression of these receptors (Fig. S1A-B and Tables S1–S2); with an increase in the proportion of GluA1 subunits and concomitant decreases in the proportion of GluA2 subunits after CCI. We propose that the change in AMPA receptor subunits, GluA1 and GluA2 might contribute towards increased neuronal activity during CCI, further suggesting that after injury Ca^2+^-permeable AMPA receptors increase at the membrane, thereby enhancing plasticity through the activation of kinases and immediate early genes [41], [42], [43].

### NMDA receptor

Following CCI, we did not observe any change in the amplitude of NMDA-mediated inward currents in small diameter neurons, but there was an increase in the percentage of NMDA-responsive neurons. This accords with a previous study showing that more primary sensory neurons and their axons express NMDA receptors after induction of peripheral inflammation [11], [44]; knockdown of NMDA receptors in DRGs reduces pain behavior in formalin test [45]. Our observation that CCI did not change the amplitude of NMDA-induced inward currents, but increased the percent of NMDA-responsive neurons by ∼200% suggested that nerve injury results in a larger population of small diameter neurons becoming NMDA responsive, rather than an increase in the activity of NMDA receptor on neurons. This data is in agreement with a previous study showing that a greater number of primary sensory neurons express NMDA receptors after induction of peripheral inflammation [11], [44]. That peripheral NMDA receptors contribute to nociception is suggested by studies showing that small diameter peptidergic neurons co-express NMDA receptors and knockdown of NMDA receptors in DRGs reduced pain behavior [45], [46]. Thus, NMDA-expressing small diameter primary sensory neurons contribute to increased nociception.

### Group I mGluR

Our results showing that CCI increased DHPG-mediated inward currents by ∼500% as well as the percentage of responsive small diameter neurons, adds further support to the idea that group I mGluRs are involved in nociception. A previous study found that almost all the DHPG-responsive DRG neurons express TRPV1 [47], which links them to nociceptive transmission. In rats with CCI, the observed increases in DHPG-induced currents are probably due to increased mGluR1 at the neuronal membrane as suggested by our western blot analysis data. Others have suggested that group I mGluRs in primary sensory neurons play a role in nociceptive processes [2], [6], [48]. For instance, intradermal injection of a group I mGluR agonist enhanced noxious heat responses, whereas antagonists attenuated formalin-induced pain [2], [49].

Group I mGluR are well known to modulate NMDA and AMPA receptor function [50], [51]. While the effect of a short exposure to DHPG would generally be expected to enhance the activity of NMDA, AMPA and KA receptors, whereas a longer exposure led to receptor desensitization and internalization [52] and secondary decrease in AMPA and NMDA receptor activity. Consistent with the latter observation, in our study, 2 hour DHPG incubation reduced both the AMPA- and NMDA-induced inward currents in naïve as well as in nerve-injured ganglia. In contrast to the results with NMDA and AMPA receptors, after DHPG incubation KA-induced inward currents increased in naïve DRGs to a level similar to that resulting from CCI. In support of our results, activation of group I mGluR is known to potentiate KA receptors activation, suggesting that KA, *but not* NMDA or AMPA receptors are likely to be differently regulated by mGluRs [52]. The lack of an effect after CCI could be because the KA currents had reached a ceiling and thus DHPG incubation could not further increase the induced currents.

### Functional implications

Considerable evidence points to peripheral sensitization in neuropathic pain [53], [54]. After nerve injury, primary sensory neurons have reduced thresholds and rheobases, show spontaneous firing causing ectopic discharges from within the DRGs [55], as well as at the injury site [56], [57]. Allthough CCI causes considerable damage to both large and small diameter axons distal to the lesion, with the large myelinated axons showing greater damage than small unmyelinated axons, there is minor or no histological changes proximal to the lesion [58], [59]. Thus, although the axons proximal to the lesion may appear morphologically unchanged, the electrophysiological alterations described above may be responsible for the intitiation or persitance of neuropathic pain. The changes in excitabilty in small diameter neurons, as oppposed to large diameter neurons, might be related to alteration of sodium channels, and it has been pointed out that changes in sodium channels are most obvious in small diameter neurons after CCI [60]. In contrast to a previously published study by Kajander et. al., [61], here, in our study, we find hyperpolarization of large diameter neurons, indicating decreased neuronal excitability. Kajander et. al. utilized a different time course for CCI (1–3 day), whereas our study recorded from DRGs after 7 days of CCI. Thus, this time course can account for observed discrepancy. Furthermore, the above study used intracellular recordings, whereas we used patch clamp, a more precise technique to record changes. Interestingly, and in agreement with our data, in that study, rheobase and membrane threshold in large diameter neurons showed no significant changes despite differences in time course of injury.

It is likely that glutamate released in the ganglion contributes to some of the changes in neuronal activity given that glutamate stimulates and sensitizes primary sensory neurons and their peripheral terminals [6], [11], [13], [62]. Sensory neurons contain abundant glutamate [1], [6] which is released peripherally [63] and most likely within the sensory ganglion. Glutamate together with ATP, CGRP and TNF-α are likely to impact nociception by their action on somatic receptors, especially after nerve injury or peripheral inflammation [64]. Our data showing increased glutamate-induced inward currents in small diameter neurons following CCI, coupled with changes in expression of membrane-bound receptors (Fig. S1C–D and Tables S3–S4) suggests that glutamate-responsive DRG neurons play a role in nociception. Our assumption that the increased response to glutamate occurs in nociceptive neurons is based both on the understanding that most small diameter DRG neurons are nociceptive and on previous reports that show co-expression of glutamate receptors and markers of nociception in small diameter DRG neurons. For instance, co-expression of group I mGluR and TRPV1 receptors [47], [65], NMDA receptors and substance P [66], as well as iGluRs and TRPV1 receptors has been reported [67]. Thus, we propose that subsets of small diameter neurons that we recorded from are probably involved in nociception.

## Conclusion

The present findings further support that peripheral glutamatergic transmission plays a role in nociceptive processes following nerve injury. Understanding the role of glutamate in nociception in the peripheral nervous system will open up avenues for developing new therapeutics aimed at reducing pain.

## Supporting Information

Figure S1 Membrane expression of glutamate receptors. Membrane expression of GluA1 (A), GluA2 (B), mGluR1 (C) and mGluR5 (D) from naïve and CCI DRG normalized to N-cadherin shows trends similar to that analyzed using membrane/total normalized ratios in the main figures.
(TIF)

Figure S2 Western blot analysis. Membrane or cytosolic protein fractions (20 µg) were separated by SDS-PAGE and transferred to PVDF membranes. Blots were incubated with antibodies to β-actin (1:7K, upper blot) and N-cadherin (1:1K, lower blot). As expected, β-actin was detected in cytosolic (C1-4) fractions, but not in membrane fractions (M1-4). N-cadherin was detected in membrane (M1-4), but not cytosolic fraction (C, lower blot), thereby confirming that no cross-contamination of cytosolic proteins were present in the membrane fraction and *vice versa*. N-cadherin and β-actin were used as loading controls to normalize for loading discrepancies. MW: molecular weight marker; KDa: kilo Dalton.
(TIF)

Figure S3 GluA1 expression in small and medium diameter neurons after CCI. Immunofluorescence showed that GluA1 immunoreactivity (GluA1-IR) was largely found in small and medium diameter DRG neurons after CCI (red arrows). In control animals, GluA1-IR was predominantly detected on small diameter neurons (red arrows). After CCI, the proportion of small and medium diameter GluA1-IR positive neurons appeared to increase in three different sections visualized. Scale bar: 50 µm.
(TIF)

Table S1 (DOCX)

Table S2 (DOCX)

Table S3 (DOCX)

Table S4 (DOCX)
