# Correction: Increased Response to Glutamate in Small Diameter Dorsal Root Ganglion Neurons after Sciatic Nerve Injury

**DOI:** 10.1371/journal.pone.0106979

**Published:** 2014-08-27

**Authors:** The PLOS ONE Staff

An affiliation for the third author is missing. See the correct affiliations for Giulia Magni here:

**2** Department of Anatomy, University of California San Francisco, San Francisco, California, United States of America

**3** Department of Surgery, University of California San Francisco, San Francisco, California, United States of America

**5** Department of Drug Discovery and Development, Italian Institute of Technology (IIT), via Morego 30, Genova, Italy
