# Supplementary figures and images for: Increased Response to Glutamate in Small Diameter Dorsal Root Ganglion Neurons after Sciatic Nerve Injury
Source: PLoS One. 2014 Apr 18;9(4):e95491. doi: 10.1371/journal.pone.0095491 (PMC3991716; doi:10.1371/journal.pone.0095491)

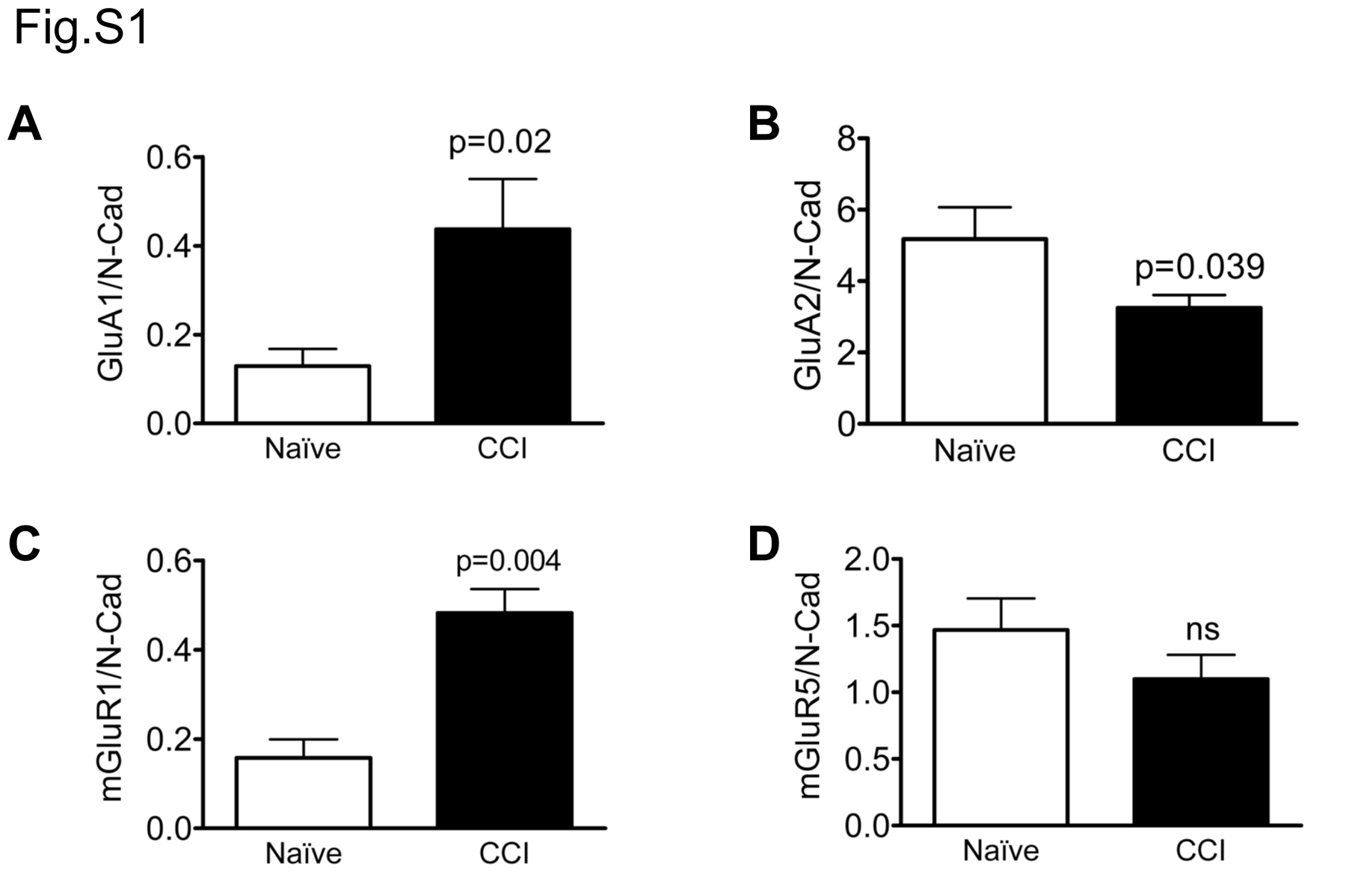

Supplement: Figure S1 — Membrane expression of glutamate receptors. Membrane expression of GluA1 (A), GluA2 (B), mGluR1 (C) and mGluR5 (D) from naïve and CCI DRG normalized to N-cadherin shows trends similar to that analyzed using membrane/total normalized ratios in the main figures. (TIF) [file pone.0095491.s001.tif]

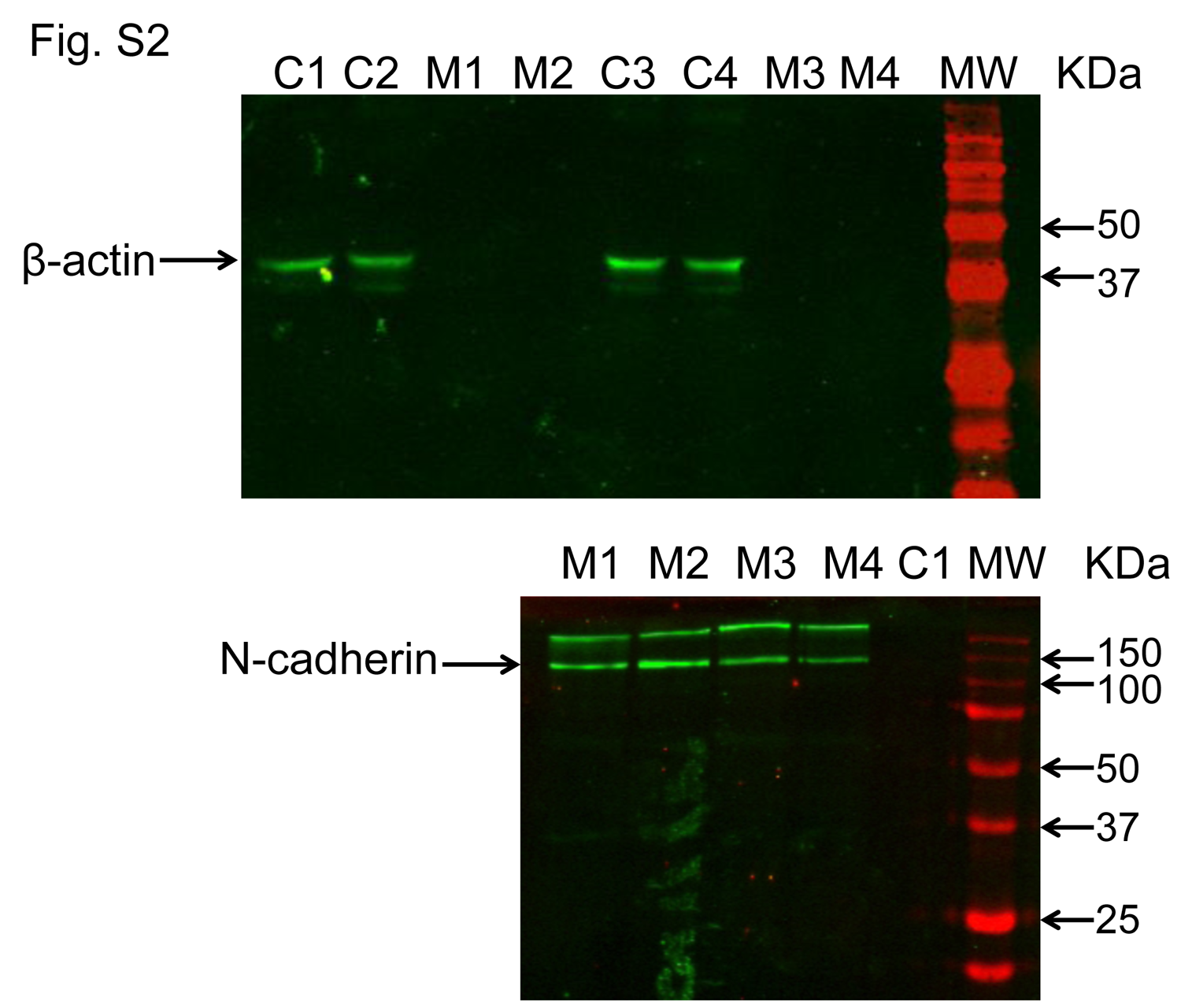

Supplement: Figure S2 — Western blot analysis. Membrane or cytosolic protein fractions (20 µg) were separated by SDS-PAGE and transferred to PVDF membranes. Blots were incubated with antibodies to β-actin (1:7K, upper blot) and N-cadherin (1:1K, lower blot). As expected, β-actin was detected in cytosolic (C1-4) fractions, but not in membrane fractions (M1-4). N-cadherin was detected in membrane (M1-4), but not cytosolic fraction (C, lower blot), thereby confirming that no cross-contamination of cytosolic proteins were present in the membrane fraction and vice versa. N-cadherin and β-actin were used as loading controls to normalize for loading discrepancies. MW: molecular weight marker; KDa: kilo Dalton. (TIF) [file pone.0095491.s002.tif]

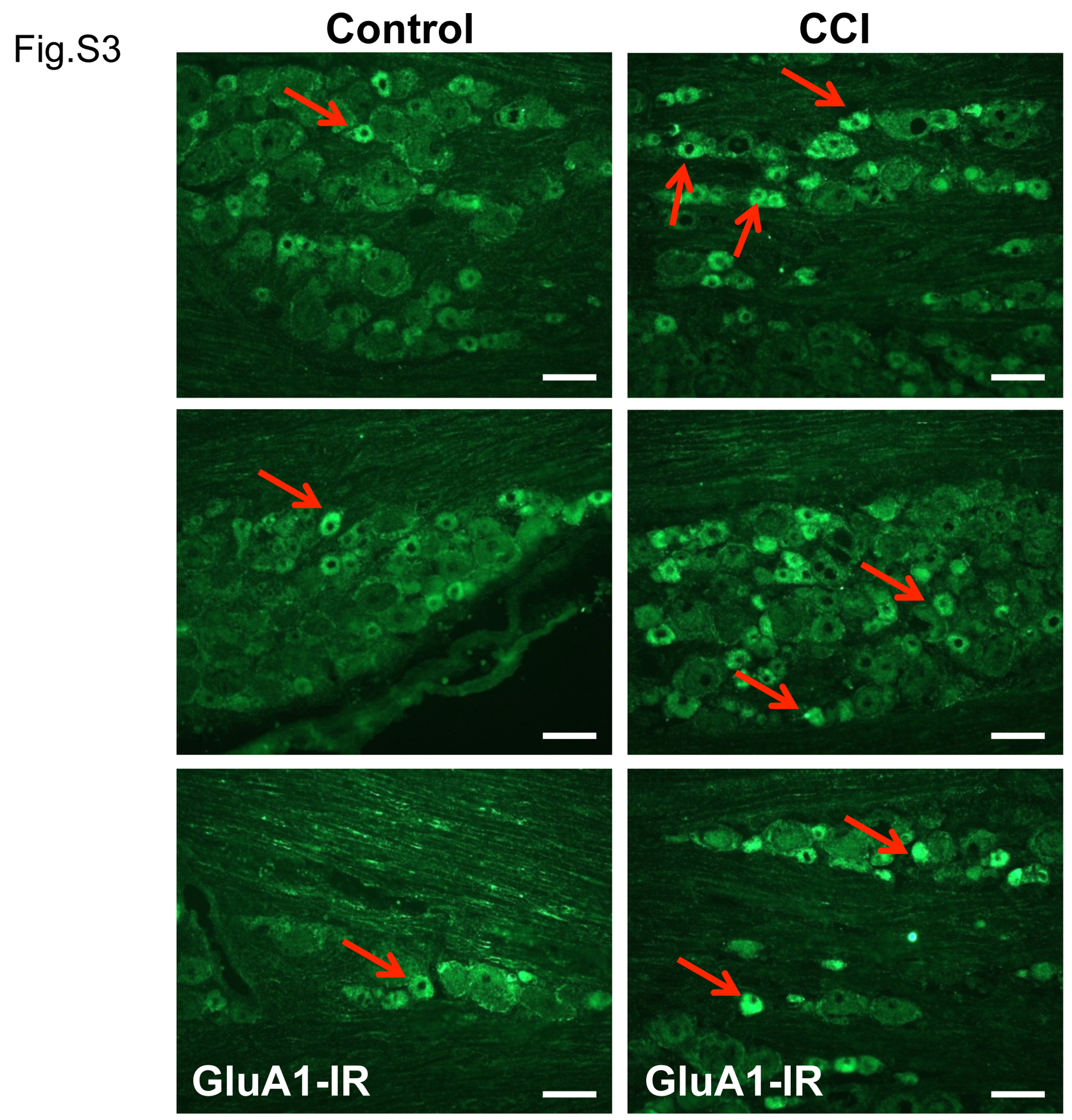

Supplement: Figure S3 — GluA1 expression in small and medium diameter neurons after CCI. Immunofluorescence showed that GluA1 immunoreactivity (GluA1-IR) was largely found in small and medium diameter DRG neurons after CCI (red arrows). In control animals, GluA1-IR was predominantly detected on small diameter neurons (red arrows). After CCI, the proportion of small and medium diameter GluA1-IR positive neurons appeared to increase in three different sections visualized. Scale bar: 50 µm. (TIF) [file pone.0095491.s003.tif]
