# Supplementary material for: Increased Response to Glutamate in Small Diameter Dorsal Root Ganglion Neurons after Sciatic Nerve Injury
Source: PLoS One. 2014 Apr 18;9(4):e95491. doi: 10.1371/journal.pone.0095491 (PMC3991716; doi:10.1371/journal.pone.0095491)
Supplement: Table S1 — (DOCX) [file pone.0095491.s004.docx]

**Table S1**. Normalized membrane protein values of GluA1 to N-cadherin from naïve and CCI DRG.

| **Naïve GluA1/N-cadherin** | **CCI GluA1/N-cadherin** |
| --- | --- |
| 0.04252158 | 0.7268448 |
| 0.2913721 | 0.2778147 |
| 0.05604164 | 0.420495 |
| 0.06124569 | 0.1501652 |
| 0.05461792 | 0.2276791 |
| 0.1523392 | 0.8204159 |
| 0.2455235 |  |
