# Supplementary material for: Increased Response to Glutamate in Small Diameter Dorsal Root Ganglion Neurons after Sciatic Nerve Injury
Source: PLoS One. 2014 Apr 18;9(4):e95491. doi: 10.1371/journal.pone.0095491 (PMC3991716; doi:10.1371/journal.pone.0095491)
Supplement: Table S2 — (DOCX) [file pone.0095491.s005.docx]

**Table S2**. Normalized membrane protein values of GluA2 to N-cadherin from naïve and CCI DRG.

| **Naïve GluA2/N-cadherin** | **CCI GluA2/N-cadherin** |
| --- | --- |
| 4.701851 | 2.325714 |
| 3.075728 | 4.782051 |
| 8.027132 | 2.562130 |
| 6.269450 | 4.644860 |
| 3.825581 | 3.509615 |
|  | 3.438776 |
|  | 2.360000 |
|  | 2.402367 |
