# Supplementary material for: Increased Response to Glutamate in Small Diameter Dorsal Root Ganglion Neurons after Sciatic Nerve Injury
Source: PLoS One. 2014 Apr 18;9(4):e95491. doi: 10.1371/journal.pone.0095491 (PMC3991716; doi:10.1371/journal.pone.0095491)
Supplement: Table S3 — (DOCX) [file pone.0095491.s006.docx]

**Table S3**. Normalized membrane protein values of mGluR1 to N-cadherin from naïve and CCI DRG.

| **Naïve mGluR1/N-cadherin** | **CCI mGluR5/N-cadherin** |
| --- | --- |
| 0.2403976 | 0.3266351 |
| 0.05684346 | 0.4687744 |
| 0.1241825 | 0.499430 |
| 0.07823998 | 0.7808209 |
| 0.0822825 | 0.4660904 |
| 0.1584512 | 0.2949181 |
| 0.3641268 | 0.5713589 |
|  | 0.451746 |
