# Supplementary material for: Increased Response to Glutamate in Small Diameter Dorsal Root Ganglion Neurons after Sciatic Nerve Injury
Source: PLoS One. 2014 Apr 18;9(4):e95491. doi: 10.1371/journal.pone.0095491 (PMC3991716; doi:10.1371/journal.pone.0095491)
Supplement: Table S4 — (DOCX) [file pone.0095491.s007.docx]

**Table S4**. Normalized membrane protein values of mGluR5 to N-cadherin from naïve and CCI DRG.

| **Naïve mGluR5/N-cadherin** | **CCI mGluR5/N-cadherin** |
| --- | --- |
| 1.200468 | 0.5815338 |
| 1.295975 | 1.922185 |
| 2.363828 | 0.4570322 |
| 0.9956842 | 0.7956021 |
| 1.478824 | 0.9474449 |
|  | 1.499287 |
|  | 1.028929 |
|  | 1.563762 |
